# A Novel Energy-Aware Distributed Clustering Algorithm for Heterogeneous Wireless Sensor Networks in the Mobile Environment

**DOI:** 10.3390/s151229836

**Published:** 2015-12-10

**Authors:** Ying Gao, Chris Hadri Wkram, Jiajie Duan, Jarong Chou

**Affiliations:** 1 College of Computer Science and Engineering, South China University of Technology, Guangzhou 510006, China; gaoying@scut.edu.cn; 2 Lawrence Berkeley National Laboratory, University of California, Oakland, CA 94612, USA; chrish.w.zhang@gmail.com; 3 Yun Nan Electric Power Test & Research Institute Group CO., Kunming 650217, China; duanzealot@163.com; 4 College of Engineering, Michigan State University, East Lansing, MI 48824, USA

**Keywords:** heterogeneous wireless sensor networks, sensor computing, distributed clustering algorithm, collaborative information processing, energy efficient, mobile sensor

## Abstract

In order to prolong the network lifetime, energy-efficient protocols adapted to the features of wireless sensor networks should be used. This paper explores in depth the nature of heterogeneous wireless sensor networks, and finally proposes an algorithm to address the problem of finding an effective pathway for heterogeneous clustering energy. The proposed algorithm implements cluster head selection according to the degree of energy attenuation during the network’s running and the degree of candidate nodes’ effective coverage on the whole network, so as to obtain an even energy consumption over the whole network for the situation with high degree of coverage. Simulation results show that the proposed clustering protocol has better adaptability to heterogeneous environments than existing clustering algorithms in prolonging the network lifetime.

## 1. Introduction

Wireless sensor networks (WSNs) are deployed in monitoring regions equipped with a large number of sensor nodes with neither partition nor infrastructure support, through the wireless self-organized network in multi-hop communication mode [1]. As a kind of new intelligent fusion of multiple disciplines, such as computer science, network technology, embedded system, calculation of multiple fields, microelectronics, sensor information acquisition and processing technology, *etc.*, wireless sensor networks can be used to sense and collect in real time all kinds of data for monitoring objects and information about the environment, through various integrated microsensors. In previous research, the sensor embedded system used to be in charge of dealing with the information, and sending the sensed information to the user terminal by multi-hop relay through a self-organized wireless communication network, so as to realize the “ubiquitous computing” concept. WSNs have created a new era for information acquisition and processing technology, and have promising application prospects in many fields, such as national defense, industry, smart city, Internet of things (IoT), cyber-physical system (CPS), radio frequency identification(RFID), *etc.* [2]. In the mobile environment, sensor networks are robust and the topology may vary as the nodes move. Providing sensor computing is however still a challenging issue, especially in heterogeneous or mobile environments.

Present studies on WSNs are mainly confined to isomorphic network fields. Isomorphism in WSNs is used to simplify all of the node sensors to be of the same structure, while ignoring the effect the heterogeneity of any node brings to the design of network and protocols [3]. At the beginning of the study, this simplified network model was used for convenience, but with the development of wireless sensors and their applications in the research fields of the depth and the promotion, the isomorphic WSN model has become competent to meet the demands of actual application scenarios. Especially in the areas of practical network application, monitoring and management of sensor nodes are mainly set for mixed environments, where two or more sensor nodes would be needed for monitoring. Therefore, in a heterogeneous world, a heterogeneous WSNs model and the actual application scenario is suitable. Heterogeneous sensor networks refer to wireless networks consisting of a variety of different types of sensor nodes. Node energies and communication capacity of heterogeneous characteristics are ubiquitous features of heterogeneous wireless sensor networks (HWSNs), this paper reports research on wireless sensor network energy optimization and a topology control algorithm appropriate for the energy and communication capacity of heterogeneous wireless sensor networks.

At present the research on heterogeneous networks is still in the initial stages of exploration, and there are still a series of unsolved problems to be solved. For example, exploring the scientific meaning of heterogeneous wireless sensor networks, and the specific content of networks in heterogeneous forms. The heterogeneous network model is at present so simple that it cannot adequately describe the actual application scenarios, thus the problem of how to construct the heterogeneous network, that involves how to design energy efficient strategies in heterogeneous networks, and thereafter improve the energy efficiency of the heterogeneous network remains unresolved. Therefore, the utilization of a large number of isomorphic WSN theories in heterogeneous networks has a profound theoretical and practical significance.

Sensor nodes organized in the form of clusters can effectively achieve energy savings in the network. Many energy-efficient routing protocols are designed based on the cluster structure [4]. In the cluster structure, throughout the network the algorithms are only executed in a cluster range without waiting for control messages. In a large network, this feature makes the scalability and robustness of localization algorithm better than the center of the algorithm execution throughout the global structure. At the same time, clustering technology is also very effective for broadcasting news and querying data, as the cluster head nodes can help broadcast messages and collect the data of users’ interest within the cluster.

At present, many domestic and foreign experts have proposed numerous different distributed clustering algorithms. The widely adopted algorithms refer to isomorphic or heterogeneous networks, which can be divided into two categories, namely those with the same composition and different composition clustering algorithms. Because of the complexity and energy configuration of network evolution, it is very difficult to design a suitable heterogeneous network energy efficient clustering algorithm. Many algorithms under the isomorphism network environment find it difficult to make full use of the heterogeneous characteristics of energy, thus low energy nodes will die earlier than high energy nodes. The proposed protocol is designed for a heterogeneous network [5,6], which lets the cluster head node and the node residual energy be directly related to the cluster head election, in the meantime retains the advantages of distributed algorithms.

In this paper, we focus on and explore in depth the nature of heterogeneous wireless sensor networks, and finally propose a novel energy-aware distributed clustering algorithm for heterogeneous wireless sensor networks (EDCA-H), which addresses the problem of finding an effective pathway for heterogeneous clustering energy. The proposed algorithm implements cluster head selection according to the degree of energy attenuation while the network is running and the degree of candidate nodes’ effective coverage on the whole network, so as to obtain an even energy consumption over the whole network for the situation with high degree of coverage.

The rest of this paper is organized as follows: a brief introduction of works related to the topic of energy saving in heterogeneous wireless sensor networks is presented in Section 2. The energy consumption model is proposed and the optimization problems are presented in Section 3. In Section 4, the design of our novel proposed protocol is described in detail. Simulation results are discussed in Section 5. Finally, conclusions are made in Section 6.

## 2. Related Works

Recently, most of the energy efficient protocols designed for heterogeneous networks are based on the clustering technique [7], which is effective for scalability and energy saving in WSNs. With special advantages related to scalability and efficient communication, clustering provides an efficient and scalable way to design and organize large-scale WSNs for data communication energy efficiency [8]. In a hierarchical architecture, higher energy nodes can be used to process and send the information while low energy nodes can be used to perform the sensing in the proximity of the target. Some of routing protocols in this group include low energy adaptive clustering hierarchy (LEACH) [9], LEACH-C [9], LEACH-M [10], energy-efficient improvement heterogeneous networks protocol (EEIHN) [11], approximate dynamic programming algorithm (ADP) [12] and efficient cluster head selection approach for collaborative data processing (CHSCDP) [13].

In those protocols, the nodes are equipped with the same energy at the beginning, but the networks cannot evolve equally for each node in expending energy, due to the radio communication characteristics, random events such as short-term link failures or the morphological characteristics of the field [14]. However, deployment of sensors in a WSN can be deterministic or random depending on the application. They can be stationary or location-aware, homogeneous or heterogeneous in nature. WSNs are more typically heterogeneous networks than homogeneous ones. The protocols should be fit for the characteristics of heterogeneous wireless sensor networks. Currently, due to the construction of a simple mathematic mode for analyzing the energy consumption and data aggregation, most clustering algorithms assume that the sensor networks are homogeneous, so these algorithms often perform poorly in heterogeneous environments.

According to the initial energy, Smaragdakis *et al.* proposed a SEP scheme [15] for a two-level heterogeneous wireless sensor network composed of two types of nodes. The advanced nodes were equipped with more energy than the normal nodes at the beginning. SEP prolonged the stability period, which was defined as the time interval before the death of the first node. However, it was not fit for the widely used multi-level heterogeneous wireless sensor networks which include more than two types of nodes. In [16], Lee *et al.* considered an event detection heterogeneous network with two types of nodes that included type-1 and type-0 node. Type-1 nodes had more battery energy than type-0 nodes. The key design features were that the energy supply was built into the nodes and the network lifetime was analyzed.

Zhu *et al.* proposed a distributed energy efficient clustering algorithm (DEEC) [17] for multi-level and two level energy heterogeneous wireless sensor networks. In this scheme, the cluster heads were selected using the probability derived from the ratio between the residual energy of each node and the average energy of the network. The nodes with high initial and residual energy had more chances of the becoming cluster heads compared to nodes with low energy. The DEEC protocol considered both two-level and multi-level heterogeneous networks. However, each node needed to possess global knowledge of the network along with its initial and residual energy in the DEEC protocol.

There are three common types of resource heterogeneity in sensor nodes: computational heterogeneity, link heterogeneity, and energy heterogeneity [18]. Computational heterogeneity means that the heterogeneous nodes have more powerful microprocessors and more memory than normal nodes. With these powerful computational resources, the heterogeneous nodes can provide complex data processing and longer-term storage features. Link heterogeneity means that the heterogeneous nodes have high-bandwidth and long-distance network transceiver than normal nodes. Link heterogeneity can provide more reliable data transmission. Energy heterogeneity means that the heterogeneous nodes are line powered, or their battery is replaceable. Among the above three types of resource heterogeneity, the most important heterogeneity is the energy heterogeneity, because both computational heterogeneity and link heterogeneity will consume more energy resources. If there is no energy heterogeneity, computational heterogeneity and link heterogeneity will have a negative impact to the whole sensor network, *i.e.*, decreasing the network lifetime. In wireless sensor networks, due to the widespread presence of heterogeneous resource nodes, many researchers have done profound work probing into HWSNs.

In [19], Kumar *et al.* proposed an energy efficient heterogeneous clustered (EEHC) scheme for heterogeneous wireless sensor networks, which particularly presented the setting, the energy model, and how the optimal number of clusters could be computed. In order to make more rational use of network energy and prolong the lifetime of multilevel heterogeneous wireless sensor networks, Peng *et al.* proposed a new heterogeneous sensor network model with heterogeneity of monitored objects and energy heterogeneity of all nodes, called energy-efficient prediction clustering algorithm (EEPCA) [20].

The low-energy nodes will die more quickly than the high-energy ones, because these clustering algorithms are unable to treat each node discriminatorily in terms of the energy discrepancy. Therefore, a heterogeneous network model in term of different initial energies was discussed in [20]. From the viewpoint of a scheme for the selection of cluster heads, models with energy heterogeneity can be separated into two categories: models with fixed cluster heads and models with periodically selected cluster heads. The former consider that the node with more initial energy will act as a cluster head directly, while the others do the basic sensing as well as data transmission in a cluster. However, in many application scenes, it is always impossible to deploy the cluster head nodes for an optimal distribution between different types of nodes, which may weaken the energy efficient strategies in HWSNs. As for the latter, all nodes will have chances to be selected as cluster heads under the LEACH protocol, and most algorithms select cluster heads based on some weighted probability of each node related to the residual energy.

In [21], Zhou *et al.* proposed a novel stable selection and reliable transmission protocol for clustering in heterogeneous wireless sensor networks, which can be applied to networks with three different kinds of heterogeneous nodes. Nodes in this protocol were divided into two common types: one performing the function of managing information and the others collecting different data (type 0, type 1). Type 1 had more complex hardware and software architectures, so they had more initial energy and greater data transfer capability, but the application of this protocol had been limited to networks with only two types of ordinary nodes. In [22], Attea and Khalil proposed a new evolutionary algorithm based on a routing protocol for heterogeneous wireless sensor networks, which had an appropriate fitness function and the intrinsic properties of clustering in mind.

## 3. Optimal Energy Consumption Model in HWSNs

### 3.1. System Model

#### 3.1.1. Definition 1

We consider a probabilistic WSN consisting of N sensors, denoted by $s_{1},s_{2},\cdots ,s_{N}$, respectively, and one sink deployed at the edge of a square area. All the sensor nodes know their location information. Furthermore, after the node is deployed, the position will not change. Each node has a unique identity and may determine the distance from the sender node based on the received signal intensity. Also, the transmission power can be adjusted freely by the nodes to save energy.

#### 3.1.2. Definition 2

In a multi-level heterogeneous network, the initial energy of nodes may be randomly distributed within a closed interval, such as $\left[E_{\min},E_{\max}\right]$, where the lower bound for the energy is $E_{\min}$ and the higher one is $E_{\max}$, determining the minimum and maximum values of the initial energy of nodes in the network. We further assume that the initial energy of each node can be defined as $\rho_{i}E_{\min}$($\rho_{i}\geq 1$). Therefore, the total initial energy of the multi-level heterogeneous network is equal to $E_{total}=\sum_{i=1}^{N}\rho_{i}E_{\min}$.

#### 3.1.3. Definition 3

If the node had not been cluster head (CH) for the last (1/*p*) rounds, the threshold $T(s_{i})$ would be a random number between 0 and 1. If the random number is less than the threshold *T* (*n*), then the node will become the cluster head in the next round and send a broadcast message. $T(s_{i})$ can be defined as follows:

(1) $$T(s_{i})=\left\{ \begin{array}{l} \frac{p}{1-p_{opt}(r\mathrm{mod}\frac{1}{p})}\times [\frac{E_{res}(i)}{E_{init}(i)}+(r_{s}div\frac{1}{p})(1-\frac{E_{res}(i)}{E_{init}(i)})],if\;s_{i}\in G\\ 0,\;otherwise \end{array}\right.$$

where *r* denotes the number of current round, and G is the set of nodes that are not being selected as the CH in recent *r* mod(1/*p_opt_*) rounds, so each node has the opportunity to become the CH. The threshold function in Equation (1) is improved actually based on LEACH, and demonstrates the bootstrap if the node has not yet been elected as cluster head node in the current 1/*p* rounds. Meanwhile, the probability value assignment for CH selection is conducted randomly to ensure that the is a suitable number of cluster heads in the network, thus to improve the coverage ratio of the whole network. However, the threshold function is focused on the application of heterogeneous wireless sensor networks [23]. For a heterogeneous network, the threshold function should be improved to be adaptive to the nodes with different initial energy.

### 3.2. Average Energy Consumption

In a hierarchical structure, the CH just works as a relay node to help network member nodes shorten their transmission distance so as to save energy consumption. For the sake of avoid making the CHs die early and causing a cascade effect in the network, a new round should begin and a new cluster structure should be built in the whole network.

Due to their performance of the additional functions of data integration and relaying messages, the CHs will consume more energy than ordinary nodes. In order to prevent the premature death of some nodes due to excessive energy overhead, all nodes of the cluster should become a CH in turn and the nodes with higher residual energy should have a greater chance. In homogeneous wireless sensor networks, the resolution will be rather simple so that all nodes have the same probability for CH election. Since CHs’ selection is random, which does not take into account the residual energy of each node or need the support of BS, we observe that the improved model adds some helpful determinacy factors during CHs’ selection that are beneficial to the cluster stabilization. In addition, in heterogeneous networks, considering that the initial energy of all nodes is not uniform and the nodes with more residual energy should have more advantages during the phrase of CH selection, we should assign different weighted probabilities to different nodes according to the initial energy so as to prolong the network lifetime. In order to derive a reasonable probability, we analyze quantitatively the energy consumption of the data aggregation and forwarding to the destination.

A radio energy model for radio hardware energy dissipation was proposed by Heinzelman [9], where the transmitter dissipated energy to run the radio electronics and the power amplifier, and the receiver dissipated energy to run the radio electronics, are given as follows:

(2) $$E_{Tx}(l,d)=\left\{ \begin{array}{l} lE_{elec}+l\varepsilon_{fs}d^{2},d<d_{0}\\ lE_{elec}+l\varepsilon_{mp}d^{4},d\geq d_{0} \end{array}\right.$$

(3) $$E_{Rx}(l,d)=lE_{elec}$$

where $E_{Tx}$ denotes the amount of energy to transmit an l bit message over distance d, $E_{Rx}$ denotes the amount of energy to receive the message, $\varepsilon_{fs}$ is the free space model of transmitter amplifier, and $\varepsilon_{mp}$ is the multipath model of the transmitter amplifier. The electronics energy $E_{elec}$ depends on factors such as the digital coding, modulation, filtering, and spreading of the signal.

In this model, both the free space ($d^{2}$ power loss) and the multipath fading ($d^{4}$ power loss) channel models were used, which depend on the distance between the transmitter and receiver. Power control can be used to invert this loss by appropriately setting the power amplifier. For example, if the distance is less than a threshold $d_{0}$, the free space model is used; otherwise, the multipath model is used.

For the sake of convenience, we do not consider the energy consumption of CH during the process of data aggregation. Suppose $E_{rec}$ is the energy consumed by CH for receiving all messages from its member nodes. $E_{toCH}$ and $E_{toBS}$ are the energy consumptions during a single-time data transmission by all nodes in the cluster and the CH respectively. Then, the total energy consumption of a cluster can be calculated as:

(4) $$E_{cluster-in}=E_{toCH}+E_{toBS}+E_{rec}$$

According to first order radio model, $E_{toCH}$ is given by:

(5) $$E_{toCH}=\int_{0}^{r_{i}}2\pi \sigma (lE_{elec}+l\varepsilon_{fs}x^{2})xdx=l\pi \sigma (E_{elec}r_{i}^{2}+\frac{1}{2}\varepsilon_{fs}r_{i}^{4})$$

where $r_{i}$ is the radius of the cluster head node for the competition, and σ($\sigma =N/M^{2}$) is the node’s density within the range.

The energy consumed by CH that forwarding data to the base station is calculated as:

(6) $$E_{toBS}=lE_{elec}+l\varepsilon_{mp}R_{i}^{4}$$

where $R_{i}$ represents the transmission radius from cluster head *i* to the base station.

Recently most algorithms are based on omnidirectional antenna technology, however, studies for using directional antennas in wireless sensor networks have gradually appeared. Compared with the omnidirectional antenna, a directional antenna model can improve the multiplexing rate of the network spatial coverage, increase the coverage area, and cost less energy consumption, *etc.* [24,25]. Once the cluster heads turn into the directional mode to communicate with a sink, Equation (3) should be changed to:

(7) $$E_{toBS}=lE_{elec}+\theta l\varepsilon_{mp}R_{i}^{4}$$

where θ is the conversion factor for energy consumption in case of switching to the directional antenna model.

In a cluster, the energy consumption of the CH for receiving messages from its member nodes is equal to:

(8) $$E_{rec}=lE_{elec}(\pi r_{i}^{2}\sigma -1)$$

Hence the average energy of the area consumed by each node is estimated as:

(9) $$\tilde{E}=\frac{E_{cluster-in}}{\;}=\frac{lE_{elec}(\pi r_{i}^{2}\sigma -1)+lE_{elec}+\theta l\varepsilon_{mp}R_{i}^{4}+l\pi \sigma (E_{elec}r_{i}^{2}+\frac{1}{2}\varepsilon_{fs}r_{i}^{4})}{\pi r_{i}^{2}\sigma }$$

By calculating the derivative of $\tilde{E}$ with respect to $r_{i}$ at zero, we can get optimal transmission radius:

(10) $$r_{i}=\sqrt[{4}]{\frac{2\theta \varepsilon_{mp}}{\pi \sigma \varepsilon_{fs}}}R_{i}$$

Substituting Equation (10) into (4), we can obtain an approximation of the total energy consumed by a single node within a cluster. According to [26], we can get the optimal cluster number $k_{opt}$ for our cluster based on the network where *N* sensor nodes are distributed randomly in a M×M sensor field as follows:

(11) $$k_{opt}=\sqrt{\frac{N}{2\pi }}\times \sqrt{\frac{\varepsilon_{fs}}{\varepsilon_{mp}}}\times \frac{M}{d^{2}}$$

Thus, the estimated value of the entire network lifetime is:

(12) $$RT=\frac{E_{total}}{k_{opt}*{\tilde{E}}_{cluster-in}}$$

In the later experiments, we consider the effect of random factors in which all nodes will not die at the same time. Then, RT in the Equation (12) is taken to be 1.5 times the estimated lifetime value.

Suppose that each node’s energy is consumed uniformly in every round, then the average energy in the round rt can be estimated as:

(13) $$\tilde{E}\left(r\right)=\frac{1}{N}E_{total}(1-\frac{rt}{RT})=\frac{RT-rt}{N*RT}E_{total}$$

In order to prolong the lifetime of the whole network, we should ensure that all nodes die at the same time. Obviously, the nodes with higher residual energy should possess a much higher average probability $p_{i}$ for cluster head selection than the other sensors, which can make the network consume energy evenly.

### 3.3. Coverage Cost Metric

In order to avoid data routing through areas sparely covered by the sensor nodes, the concept of coverage cost was originally introduced into DAPR as a routing metric by Perillo [27]. Since then, there have been lots of studies focusing on analyzing the coverage cost metric to prolong the lifetime of a network.

In the Coverage Preserving Protocol, Nghiem proposed a cluster-head selection algorithm based on the coverage cost or normalized sensing coverage area of each sensor node [28]. The sensing area only covered by a node is defined as $\phi_{0}$, and its neighbors can be defined as ϕ(m):

(14) $$\phi (m)=\phi_{0}+\sum_{i=0}^{\infty }\frac{\phi_{i}}{i+1}$$

where $\phi_{i}$ is the sensing area of the adjacent node i.

For the simplest example, ϕ(m) is calculated as:

(15) $$\phi (m)=\phi_{0}+\frac{\phi_{1}}{2}=(1-\frac{S(m,n)}{\pi R^{2}})+\frac{S(m,n)}{2\pi R^{2}}=1-\frac{S(m,n)}{2\pi R^{2}}$$

where $\phi_{1}$ is sensing area covered by node m and node n, S(m,n) is the overlap rate corresponding to those two nodes. In fact, the sensing range of a specific node m is likely to overlap with several nodes, thus the calculations become complicated and one needs location information for all the nodes. They approximate all neighbor nodes as an equivalent node with an equivalent distance to the desired node m. The estimation of this distance is based on the energy consumption of transmitting and receiving beacon messages to/from all the neighbor nodes.

To calculate the coverage cost, every node m firstly calculates its coverage cost and estimates its normalized effective sensing area in the initialization and set-up phase. Since it is too complicated to calculate the exact value of ϕ(m), we proposes an approximate approach based on the amount of energy consumption to transmit and receive beacon messages.

The specific node m within a radius of R sends beacon messages to neighbors that are within the range of 2R. The transmission energy $E_{Tran}$ and receiving energy $E_{Reci}$ are calculated respectively by:

(16) $$\left\{ \begin{array}{l} E_{Tran}=E_{Sense}+PL(2R)\\ E_{Reci}=10\log_{10}\frac{\sum_{i=0}^{K}10^{\frac{\alpha_{i}}{10}}}{Set(m)} \end{array}\right.$$

where R is the sensing radius of node *m*. $E_{Sense}$ represents the sensitivity of the radio receiver, and function PL denotes as the propagation loss for a distance. Assume that there are Set(m) neighbor nodes responding to beacon messages. Besides, $\alpha_{i}$ is the received signal energy level of node i.

Then, the equivalent distant $R^{\prime }$ is approximated by:

(17) $$R^{\prime }=2R10^{(E_{Tx}-E_{Rx})/10\eta }$$

where η denotes as the path loss exponent.

With $\Delta =R^{\prime }/2R$, the equivalent normalized overlapping area of node m is obtained:

(18) $$S(m)=\frac{2[\cos^{-1}(\Delta )-\Delta \sqrt{1-\Delta^{2}}]}{\pi }$$

According to Equation (14), ϕ(m) can be given as $\phi (m)=1-\frac{S(m)}{2}$, and we can obtain that the less ϕ(m), the higher the probability of node m becomes the cluster head.

## 4. Optimization of Cluster Head Selection

### 4.1. Competition Probability of CHs

The cluster head nodes need not only collect and process data detected by cluster member nodes, but also are responsible for forwarding data, thus the reasonable selection of cluster heads is very important. In addition, the influence of the number of cluster head nodes on the network lifetime is crucial [29]. If the cluster head node is too small, the covered cluster region will be greater, the longer the distance from member nodes to the cluster head will be, and the greater the energy consumption of data transmission. The amount of clusters is too large if the energy consumption of cluster head nodes is greater than that of the non-cluster head nodes, and the energy consumption of the whole network would be large. In this paper, taking the average power of $\tilde{E}\left(r\right)$ as a reflection of the residual energy and node energy, in addition to considering node coverage rate, one will obtain:

(19) $$p_{i}=p_{opt}[1-\frac{\tilde{E}(r)-E_{res}(i)}{\tilde{E}(r)}](1-\frac{1}{\phi (m)})$$

when the residual battery power of nodes is larger than the average energy, the average selection probability $p_{i}$ of a node will increase the proportion of the corresponding value $p_{opt}$. Otherwise, the average selection probability $p_{i}$ of node will be smaller than $p_{opt}$.

### 4.2. Heterogeneous Node Optimization

The SEP protocol [15] is a heterogeneous network using cluster head selection algorithm. In the two stages of heterogeneous sensor networks, containing only advanced nodes and normal nodes, we assume that $E_{0}$ denotes the initial energy of normal nodes, γ denotes the proportion of advanced nodes, δ denotes the initial energy of advanced nodes compared to the initial energy of normal nodes. The initial energy of γN nodes is $E_{0}\rho$ in the N nodes, and the initial energy of the other nodes is $E_{0}$. SEP sets different weighted probabilities for advanced nodes and normal nodes, respectively:

(20) $$\left\{ \begin{array}{l} p_{adv}=\frac{p_{opt}}{1+(\rho -1)\gamma }\\ p_{nor}=\frac{p_{opt}\rho }{1+(\rho -1)\gamma } \end{array}\right.$$

In general, the average probability $p_{i}$ determines the cluster head rotation cycle $n_{i}$ and threshold $T(s_{i})$ of node $s_{i}$. In Equation (20), $p_{opt}$ is the reference value of $p_{i}$. In an isomorphic network, all nodes have the same initial energy. Therefore, nodes are using the same $p_{opt}$ as the reference point, an each node of the cluster head rotation cycle of the reference valueis 1/$p_{opt}$. When the network is heterogeneous, nodes should be assigned different initial energies and use different reference values. In the two stage heterogeneous network, we select the weighted probability as the node reference value that is given in Equation (19), and then replace the $p_{opt}$ of Equation (20), then the following equation will be obtained:

(21) $$p_{i}=\left\{ \begin{array}{l} \frac{p_{opt}}{1+(\rho -1)\gamma }[1-\frac{\tilde{E}(r)-E_{res}(i)}{\tilde{E}(r)}](1-\frac{1}{\eta (m)}),\;if\;s_{i}\;is\;the\;normal\;node\\ \frac{p_{opt}\rho }{1+(\rho -1)\gamma }[1-\frac{\tilde{E}(r)-E_{res}(i)}{\tilde{E}(r)}](1-\frac{1}{\eta (m)}),\;if\;s_{i}\;is\;the\;advanced\;node \end{array}\right.$$

We can extend this model to the case of a multi-level heterogeneous network. Through the use of the weighted probability of Equation (5) instead of the $p_{opt}$ in Equation (8), one can obtain:

(22) $$p_{i}=\frac{p_{opt}N(1+\rho_{i})}{\left(N+\sum_{i=1}^{N}\rho_{i}\right)}[1-\frac{\tilde{E}(r)-E_{res}(i)}{\tilde{E}(r)}](1-\frac{1}{\eta (m)})$$

In which, $I_{i}=(\sum_{i=1}^{N}\rho_{i})/p_{opt}\rho_{i}$ denotes the basic rotation cycle of $s_{i}$, which is termed as the reference period. The corresponding $I_{i}$ is different if the initial energy is different, $n_{i}=1/p_{i}$. It can be inferred from Equation (22) that the rotation cycle $n_{i}$ of each node fluctuates around the reference cycle $I_{i}$ according to the change of residual energy. If $\tilde{E}(r)<E_{res}(i)$, then $n_{i}<I_{i}$. This means that nodes with higher initial and residual energy than the low energy nodes have greater chances of becoming the cluster head nodes, so that the network energy can be evenly distributed in the evolution process.

### 4.3. Node Sleep Scheduling Algorithm

The network lifetime is divided into different time periods, and each time section is divided into a node self-organizing scheduling stage and a working stage. Node selection is responsible for monitoring the target object in the scheduling phase, and the cluster head nodes target data acquisition at the cluster for forwarding to the sink node, while other nodes enter a sleep state. First, they perform as a neighbor node and broadcast a Protocol Independent Multicast (PIM) message. In order to avoid multiple nodes and sending the PIM message leading to channel conflict and message loss, in sending the PIM message we wait for a random time. The PIM message contains a node ID, node residual energy, location and other members in the cluster node ID and other information. A redundant coverage judgment needs to consider the coverage of all neighbor nodes. In order for this stage to have a minimum energy overhead, it just makes sure of the transmission distance to its one-hop neighbor nodes when sending a PIM message. In this way, each node can only receive data from one-hop neighbor nodes which send the PIM message. After collecting all the infor timeafter mation of neighbors, if we judge there is redundant coverage nodes, each node before dormancy waits for a period of time in order to avoid also being judged themselves as redundant nodes and the emergence of coverage blind spots. This waiting rule is implemented by setting the timer for a certain period of time after the end of the transition to a dormant state.

Therefore, this paper introduces a node residual energy balance priority scheduling mechanism. For the cover-relevant nodes s and V, if the initial energies are $E_{u}$ and $E_{v}$, then the corresponding residual energies are $E_{res}(u)$ and $E_{res}(v)$. We set the timer as follows:

(23) $$T_{off}(u)=\mu (1-|E_{u}-E_{res}(u)|/E_{res}(u))t_{u}\text{ and }T_{off}(v)=\mu (1-|E_{v}-E_{res}(v)|/E_{res}(u))t_{v}$$

where $t_{i}$ is the current time of node i, i=s,v, and μ is the adjust parameters of system. The specific node sleep scheduling mechanism is given as follows:

(1) if $T_{off}(v)\neq T_{off}(u)$, the timer is as a node sleep standard.
(2) if $T_{off}(v)=T_{off}(u)$, node ID is as priority dormancy mechanism.

In order to realize the activation of residual energy balance between nodes, reduce the activation of the nodes in a cycle exhausted energy probability, the value of $T_{off}(i)$ should be set. If $T_{off}(v)$ is equal to $T_{off}(u)$, then according to their ID number they will decide whether to sleep to not destroy the residual energy discussed in the above equilibrium principle. Judging whether a node has redundant coverage, the average residual energy of the related node residual energy is closer, while its dormant waiting time is longer, and the probability of dormancy is smaller. Each redundant node must send a source-specific multicast (SSM) message to the cover before the related nodes sleep. If it receives the SSM message of other nodes before the end of timing, the node will workas an active node, and mark the sending node dormancy. Finally, the node is removed from active neighbor list. In order to further improve the network coverage quality, the node cannot immediately fall into hibernation, so as to continue to wait for the preparation period of time known as the dormancy. If the node receives the SSM message in the dormant preparation period, it should judge whether it can sleep, otherwise, it immediately turns into an active node. In order to save energy, all nodes should fall into the sleep preparation period regardless of the final conversion to whatever state. Each node determines its state in the finish node self-scheduling, and will maintain a period of time until the system satisfies the preset coverage quality. Then, the sleep nodes are wakening up, and a new round of neighbor discovery and state scheduling is started. The node self scheduling algorithm is described as follows:

Step 1. Send a PIM message, and collect information from neighbor nodes;

Step 2. To determine whether redundant nodes are possible. If there is a redundant node, then there is a delay for a period $T_{off}(i)=\mu (1-|E_{i}-E_{res}(i)|/E_{res}(i))t_{i}$. Otherwise, the node is marked as an active node;

Step 3. The possible redundant nodes monitor the communication channel in the delay period $T_{off}(i)$. If a SSM message is received, then mark the sending node dormancy, and delete it from the active node list. The node will re-judge whether it is still a possible redundant node. If it is a redundant node, it continues to monitor the communication channel, otherwise, it is immediately marked as an active node;

Step 4. At the end of the delay $T_{off}(i)$, it sends a SSM message to the related neighbor nodes, and enters into the dormant preparation period, and continues to monitor the communication channel. During this period DCHSby if a SSM message is received, the sending node dormancy and mark from the active sending nodes in a node list is deleted. If it is a redundant node, it continues to monitor the communication channel, otherwise, it is immediately marked as an active node;

Step 5. At the end of the sleep preparation period, the transfer to a dormant state will be immediately started.

## 5. Simulation and Results Analysis

In this section, we perform simulations in MATLAB to evaluate the performance of both EDCA-H and other protocols. There are 100 sensor nodes randomly distributed in the 100 m × 100 m area. The base station is located at the top of the sensing area with the distance of 75 m. The base station and all the sensor nodes are assumed to be fixed. None of the sensor nodes are equipped with devices for detecting their own locations and their own location may be estimated with some localization algorithms. All the sensor nodes are homogeneous with limited energy. In the simulations of this section, let $E_{elec}$ = 50 nJ/bit, $E_{fs}$ = 10 pJ/bit/m^2^, $E_{mp}$ = 0.0013 pJ/bit/m^4^, θ=0.75 and η=2.5. In order to compare with other protocols, the impact of collisions and radio channels from signal interference caused by random factors is neglected. We will compare the performance of EDCA-H, LEACH-C [9], SEP [15], DCHS [23] and other protocols.

**Figure 1:**
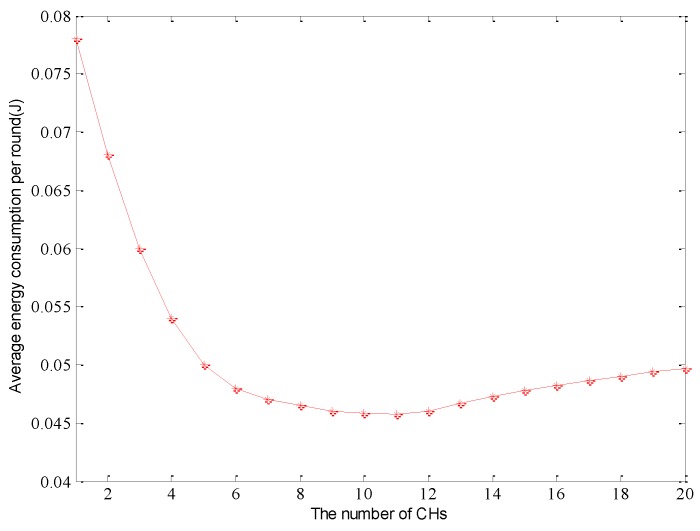
The relationship between the number of CHs and average energy consumption in each round.

As the parameter ρ increases, the gap of the initial energy among the different type of nodes is large, and it leads to sensors with a high level of energy being superior during the CH competition phase. After the formation of the clusters, a cluster head usually consumes much more energy than non-cluster nodes. Due to the energy divergence in heterogeneous wireless sensor networks, it is necessary to increase the number of clusters to balance the residual energy of different types of cluster head. Hence, this will prolong the network lifetime. Figure 1 shows the relationship between the number of clusters and the total energy consumption in each round. From the experimental results, we can observe that the cluster heads will all display high initial node energy with the growth of the parameter α. In Figure 1, the number of cluster heads will tend to the same value and the number of clusters of the optimization theory can be obtained as 11. This confirms that the optimal number of cluster heads can achieve the objective of minimizing network energy consumption in each round.

**Figure 2:**
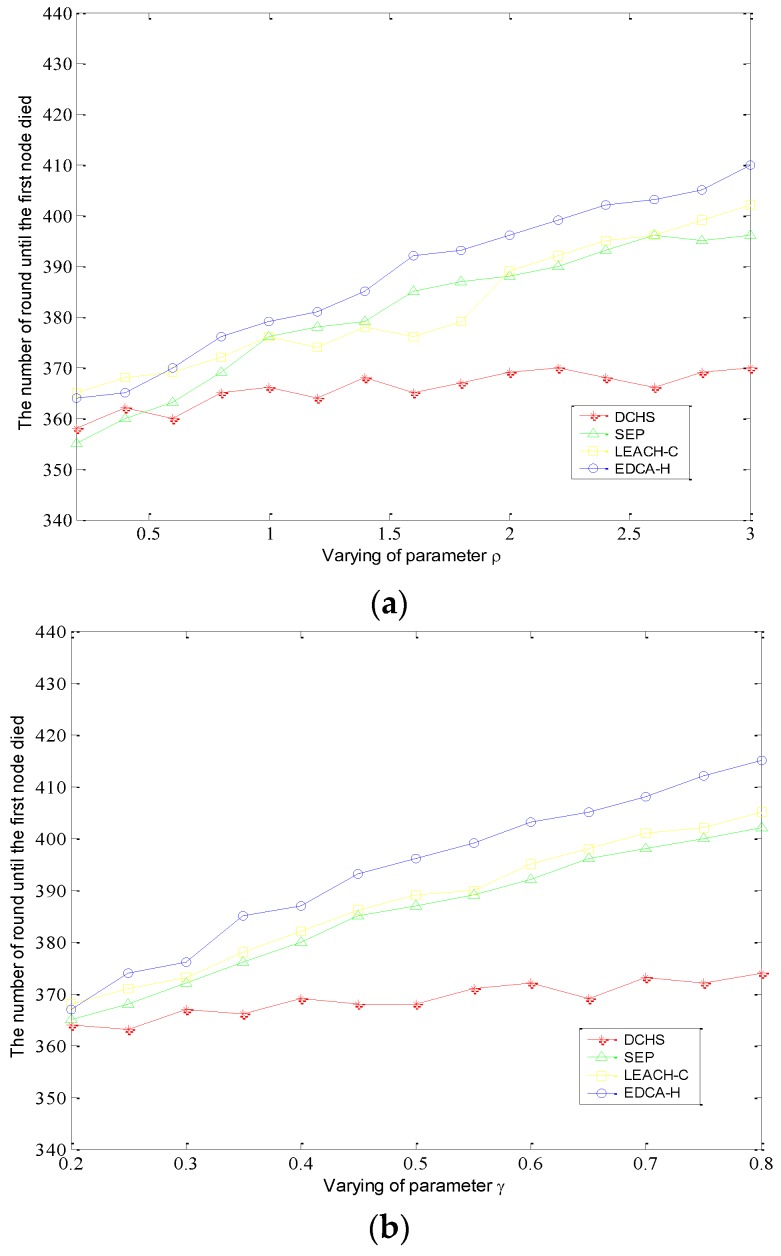
First node death when parameters ρ and γ are varied. (**a**) The value of ρ varies from 0.2 to 3; (**b**) The value of γ varies from 0.2 to 0.8.

**Figure 3:**
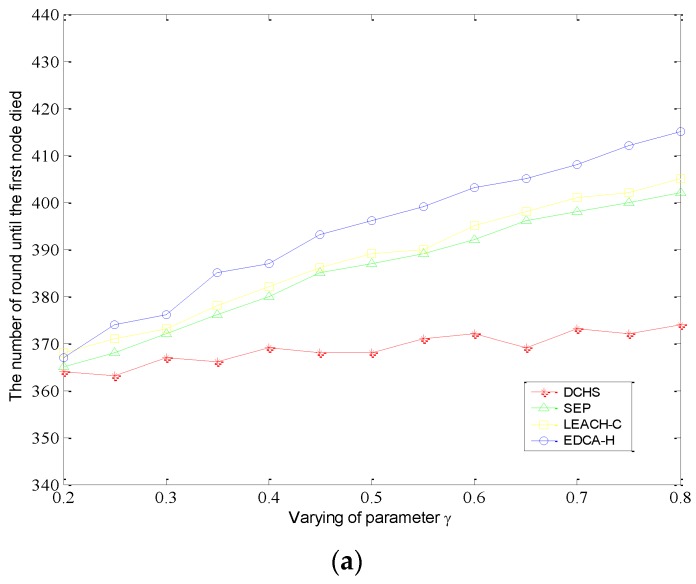

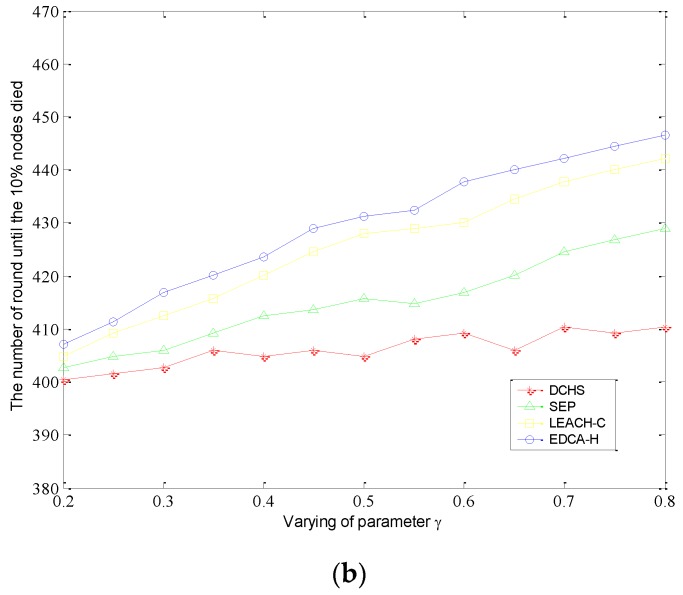
10% nodes die when parameter δ and γ are varying. (**a**) The value of ρ varies from 0.2 to 3; (**b**) The value of γ varies from 0.2 to 0.8.

For two-level heterogeneity, we evaluate the performance of the different protocols by varying the parameters γ and ρ. Figure 2 shows the comparison of rounds until the first node died as γ increases from 0.2 to 0.8 and ρ increases from 0.5 to 5, respectively. As shown in the Figure 2, DCHS performs too smoothly indicating that DCHS does not make good use of the additional energy owing to the setting of parameters ρ and γ. This performance demonstrates that it is not fit for heterogeneous environments, due to the fact it does not take into account the difference in energy and treats all nodes equally, regardless of whether their initial energy is the same or not. For the SEP protocol, we obtain the results that the stable period ratio is more than DCHS by about 25%. Although each node needs to know the residual energy of all nodes around the network for LEACH-E and this is not easy to achieve, t its performance is good in a heterogeneous network. Figure 3 shows that the stabilization period of LEACH-E exceeds that of SEP by about 10%. This is because LEACH-C takes into account the residual energy of the nodes during the selection of cluster heads in the set-up phase. EDCA-H is also a clustering protocol that considers of nodes’ residual energy like LEACH-C, besides the coverage ratio of the nodes around the network, so it exhibits better performance than other protocols, and especially when α changes, the stabilization period in EDCA-H is increased by nearly 20 percent compared to LEACH-C.

Furthermore, we also examine the number of rounds until 10% of the nodes run out of energy. Before these nodes die, the data transmission to the base station can be of high quality and reliability. Figure 3 shows that EDCA-H can achieve a long stabilization period. 

Meanwhile, it can be noted that EDCA-H can obtaina stabler period than LEACH-C and SEP with the increase of ρ and γ. For multi-level heterogeneous networks, we define that the nodes’ initial energy is distributed randomly in the range of $\left[E_{0},3E_{0}\right]$, where normal nodes are equipped with initial energy $E_{0}$. In addition, the total initial energy of the different level heterogeneous networks remains similar in each scenario.

**Figure 4:**
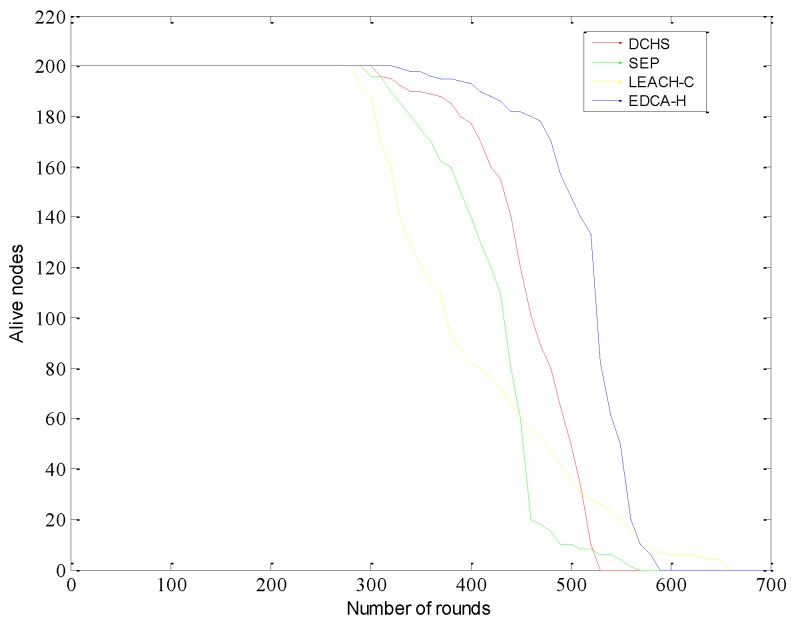
Number of active nodes over time.

Figure 4 shows the comparison of the number of active sensor nodes in each round. A sensor node is considered as active if its residual energy is not zero and also can communicate with the adjacent nodes within its communication range. Sometimes a few CHs die quickly due to improper load balancing. As a result, a few sensor nodes are unable to find any CH within their range, though the sensor nodes still may have some residual energy. It can be observed that our proposed method can obtain a longer lifetime than other protocols. 

**Figure 5:**
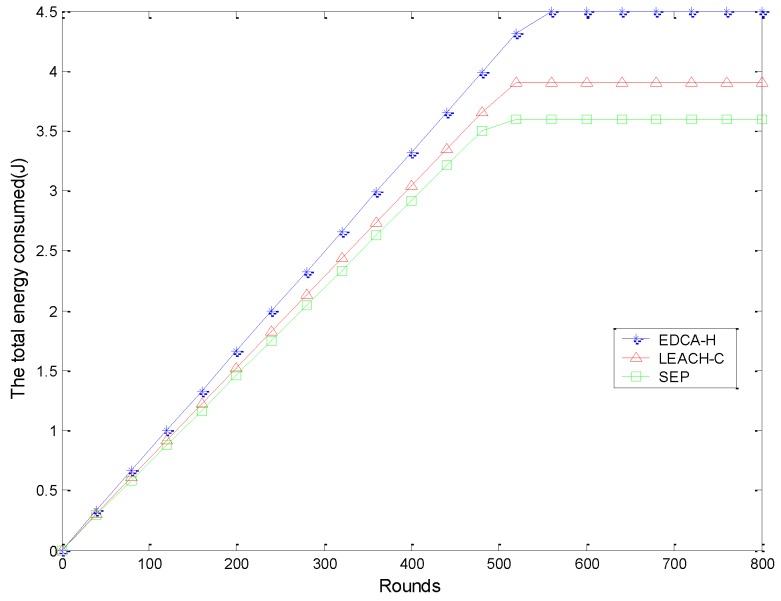
The number of messages received by base station over time.

As the LEACH algorithm does not consider the difference of nodes’ initial energy in heterogeneous wireless sensor networks, it makes good use of additional energy from the advanced nodes. The stabilization period is very short in DCHS and the nodes died according to a fixed rate. We find that our algorithm achieves the highest lifetime compared with the other approaches. Specifically, our algorithm improves the lifetime by 17.83% compared with DCHS and 8.79% compared with SEP. The improvement is less significant compared with SEP because SEP divides the area into several sectors in order to minimize the transmission distance from sensors to CHs as well as to balance the cluster size. However, EDCA-H can outperform LEACH-C in terms of effective amount of data as shown in Figure 5.

**Figure 6:**
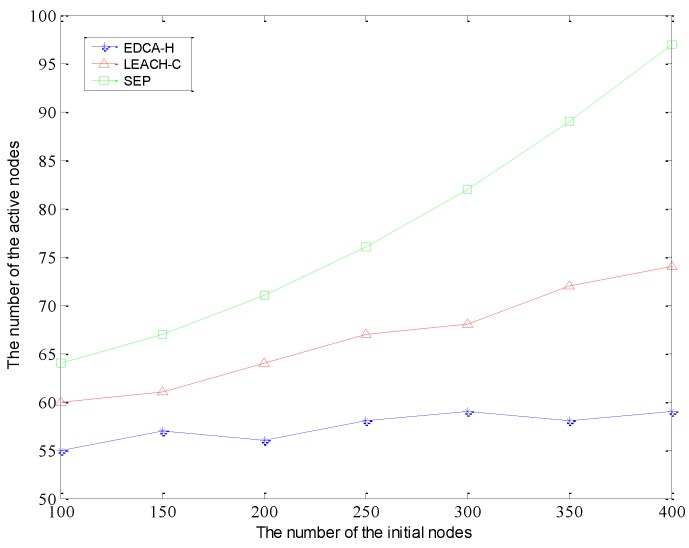
The number of initial sensors *vs.* the number of active sensors.

In addition, we investigate the number of active nodes under the condition of different network size as shown in Figure 6. When the number of nodes increases in the initial deployment, the number of active nodes fluctuates within a narrow range for EDCA-H. This is because the node’s sensing distance and the area being monitored are fixed, and the number of active nodes for covering the entire network should not vary much. However, due to the lack of an effective mechanism which can make most of redundant nodes around the border of clusters turn to a sleep state, the number of border nodes increases as the number of nodes in the initial phase increases. Then, relatively many more redundant nodes are distributed around the edge of clusters consume more energy than EDCA-H.

**Figure 7:**
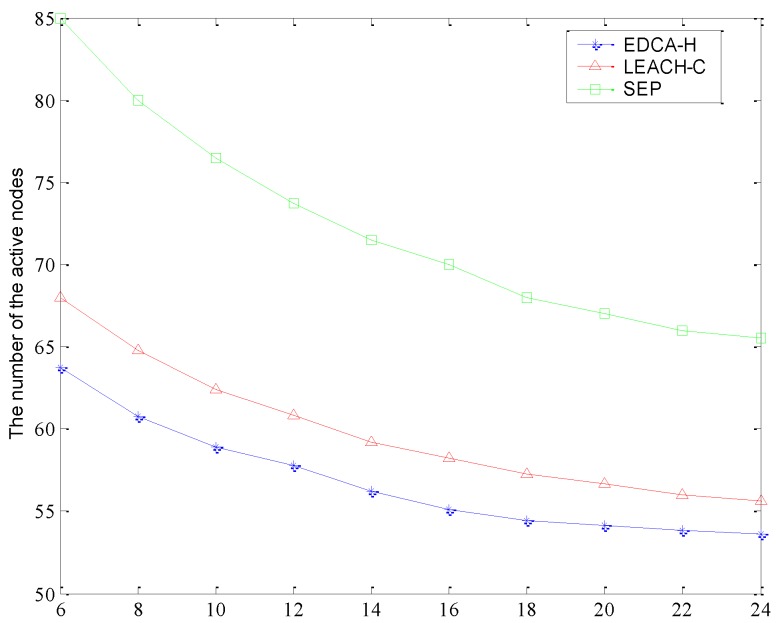
The sensing range *vs.* number of active sensors.

As can be seen from Figure 7, the number of active nodes demonstrates different degrees of decline as the sensing distance increases to ensuring a high coverage ratio for the whole network. This is because the larger the distance between sensing nodes, the greater the coverage of a single node is. Moreover, to preserve the network coverage, a smaller number of dead nodes is not as as important as a more even distribution of dead nodes. EDCA-H can make use of a rather smaller number of active nodes to achieve a highly valid coverage area. We also see that the number of active nodes in SEP decreases rapidly when the nodes’ sensing distance increases. In the scenario of 100 sensors initially distributed in the network, the proportion of active nodes in EDCA-H is less than LEACH-C by about 8.37%, and also less than SEP by about 47.5%. However, in the scenario where 300 sensors are deployed, the average number of active nodes of EDCA-H decreases sharply to 58, which is much less than the 17.5% of LEACH-C and 45.2% of SEP. Obviously, the greater the distance of the nodes’ sensing, the superiority of EDCA-H algorithm is more obvious.

## 6. Conclusions

As a heterogeneous wireless sensor network energy consumption is not uniform, and the energy utilization rate is low. This paper studied the energy consumption of a heterogeneous clustered sensor network, and proposed a new energy efficient routing algorithm for clustering in such networks. The proposed method selects the cluster head node according to the energy of the nodes during the running of the network, so as to achieve high coverage. In the future, with the adoption of mobile sensor nodes, we will study whether the balance of energy consumption among nodes can be achieved through density regulation, which aims at maximizing network lifetime.

## References

[1] Rani S., Talwar R., Malhotra J., Ahmed S.H., Sarkar M., Song H. (2015). A Novel Scheme for an Energy Efficient Internet of Things Based on Wireless Sensor Networks. Sensors.

[2] Ge X., Huang X., Wang Y., Chen M., Li Q., Han T., Wang C.-X. (2014). Energy-Efficiency Optimization for MIMO-OFDM Mobile Multimedia Communication Systems with QoS Constrains. IEEE Trans. Veh. Technol..

[3] Alanazi A., Elleithy K. (2015). Real-Time QoS Routing Protocols in Wireless Multimedia Sensor Networks: Study and Analysis. Sensors.

[4] Xu J., Yang G., Chen Z.-Y., Chen L., Yang Z. Performance analysis of Data Aggregation Algorithms in Wireless Sensor Networks. Proceedings of the International Conference on Electrical and Control Engineering.

[5] Jarry A., Leone P., Nikoletseas S., Rolim J. (2011). Optimal Data Gathering Paths and Energy-balance Mechanisms in Wireless Networks. Ad Hoc Netw..

[6] Li J., Mohapatra P. (2007). Analytical Modeling and Mitigation Techniques for the Energy Hole Problem in Sensor Networks. Pervasive Mob. Comput..

[7] Koucheryavy A., Salim A. Cluster-based Perimeter-coverage Technique for Heterogeneous Wireless Sensor Networks. Proceedings of the 2009 International Conference on Ultra Modern Telecommunications and Workshops.

[8] Abusaimeh H., Yang S.H. (2009). Dynamic Cluster Head for Lifetime Efficiency in WSN. Int. J. Autom. Comput..

[9] Heinzelman W., Chandrakasan A., Balakrishnan H. Energy-efficient Communication Protocol for Wireless Microsensor Networks. Proceedings of the 33rd Hawaii International Conference on System Sciences.

[10] Heinzelman W.R., Chandrakasan A.P., Balakrishnan H. (2002). Application-specific Protocol Architecture for Wireless Microsensor Networks. IEEE Trans. Wirel. Commun..

[11] Tuah N., Ismail M., Jumari K. (2012). Energy-efficient Improvement for Heterogeneous Wireless Sensor Networks. Inf. Technol. J..

[12] Xiang X., Lin C., Chen X. (2014). Energy-Efficient Link Selection and Transmission Scheduling in Mobile Cloud Computing. IEEE Wirel. Commun. Lett..

[13] Qiang Y., Pei B., Wei W., Li Y. (2015). An Efficient Cluster Head Selection Approach for Collaborative Data Processing in Wireless Sensor Networks. Int. J. Distrib. Sens. Netw..

[14] Dumbrava A., Kacimi R., Dhaou R., Beylot A.-L. Proportion Based Protocols for Load Balancing and Lifetime Maximization in Wireless Sensor Networks. Proceedings of the Ad Hoc Networking Workshop of the 9th IFIP Annual Mediterranean.

[15] Smaragdakis G., Matta I., Bestavros A. SEP: A Stable Election Protocol for Clustered Heterogeneous Wireless Sensor Networks. Proceedings of the Second International Workshop on Sensor and Actor Network Protocols and Applications.

[16] Lee H.Y., Seah K.G., Sun P. Energy Implications of Clustering in Heterogeneous Wireless Sensor Networks-an Analytical View. Proceedings of the 17th Annual IEEE International Symposium on Personal, Indoor and Mobile Radio Communications.

[17] Zhu Q., Wang M., Qing L. (2006). Design of a Distributed Energy-efficient Clustering Algorithm for Heterogeneous Wireless Sensor Networks. Comput. Commun..

[18] Yarvis M., Kushalnagar N., Singh H. Exploiting Heterogeneity in Sensor Networks. Proceedings IEEE the 24th Annual Joint Conference of the IEEE Computer and Communications Societies (INFOCOM 2005).

[19] Kumar D., Aseri T.C., Patel R.B. (2009). EEHC: Energy Efficient Heterogeneous Clustered Scheme for Wireless Sensor Networks. Comput. Commun..

[20] Peng J., Liu T., Li H., Guo B. (2013). Energy-Efficient Prediction Clustering Algorithm for Multilevel Heterogeneous Wireless Sensor Networks. Int. J. Distrib. Sens. Netw..

[21] Zhou H., Wu Y., Hu Y., Xie G. (2010). A Novel Stable Selection and Reliable Transmission Protocol for Clustered Heterogeneous Wireless Sensor Networks. Comput. Commun..

[22] Attea B.A., Khalil E.A. (2012). A New Evolutionary Based Routing Protocol for Clustered Heterogeneous Wireless Sensor Networks. Appl. Soft Comput. J..

[23] Handy M.J., Haase M., Timmermann D. Low Energy Adaptive Clustering Hierarchy with Deterministic Cluster-head Selection. Proceedings of the 4th International Workshop on Mobile and Wireless Communications Network.

[24] Shu W., Wang W., Wang Y. (2014). A Novel Energy-efficient Resource Allocation Algorithm Based on Immune Clonal Optimization for Green Cloud Computing. EURASIP J. Wirel. Commun. Netw..

[25] Liu J., Sun Q., Li S. (2012). Topology Control Algorithm Based on Directional Antenna in Wireless Ad Hoc Networks. J. Northeast. Univ. (Nat.Sci.).

[26] Halke R., Kulkarni V.A. (2012). En-LEACH Routing Protocol for Wireless Sensor Network. Int. J. Eng. Res. Appl..

[27] Perillo M., Heinzelman W. DAPR: A Protocol for Wireless Sensor Networks Utilizing an Application-based Routing Cost. Proceedings of the IEEE Wireless Communications and Networking Conference.

[28] Nghiem T.P., Kim J.H., Lee S.H. (2009). A Coverage and Energy Aware Cluster-head Selection Algorithm in Wireless Sensor Networks. Lect. Notes Comput. Sci..

[29] Wei W., Qi Y. (2011). Information Potential Fields Navigation in Wireless Ad-Hoc Sensor Networks. Sensors.

